# Human Endonuclease G Preferentially Cleaves Oxidatively Damaged DNA

**DOI:** 10.1021/acs.biochem.5c00669

**Published:** 2026-03-19

**Authors:** Wen-Ting Lu, Yi-Ping Chen, Wei-Zen Yang, Hanna S. Yuan, Jason L. J. Lin

**Affiliations:** † Institute of Molecular Biology, 38017 Academia Sinica, Taipei 11529, Taiwan; ‡ Graduate Program in Life Sciences, Department of Life Sciences, National Central University, Taoyuan 320317, Taiwan

## Abstract

Endonuclease G (EndoG) is a conserved endonuclease implicated in mitochondrial DNA (mtDNA) replication, maintenance of mtDNA integrity under oxidative stress, and the removal of nuclear and paternal mtDNA during apoptosis and early embryogenesis. Despite its biological significance, the substrates targeted by EndoG and its cleavage preferences remain unclear. Here, we characterize human EndoG (hEndoG) across diverse nucleic acid substrates, including single-stranded DNA (ssDNA), double-stranded DNA (dsDNA), nicked and gapped dsDNA, modified dsDNA containing 8-oxoguanine (oxoG-DNA) and hydroxymethylated cytosine (5hmC-DNA), single-stranded RNA (ssRNA), and RNA/DNA hybrids. We show that hEndoG binds most of these substrates with only modest differences in affinity (∼10-fold), yet displays a particularly strong preference for cleaving oxidatively damaged DNA, including nicked and gapped dsDNA, and oxoG-DNA. Notably, hEndoG preferentially cleaves the strand opposite the gapped or nicked site, and it targets the complementary strand to the modified base in oxoG-DNA and 5hmC-DNA. Our structural modeling of hEndoG bound to ssDNA and dsDNA indicates that ssDNA is a favored substrate because its flexibility allows kinked conformations that position the scissile phosphate near the catalytic Mg^2+^ in the His-Me finger motif. Together, these findings support a critical role for hEndoG in preserving mitochondrial genome integrity under conditions of oxidative stress by selectively targeting and removing oxidatively damaged DNA.

## Graphical abstract

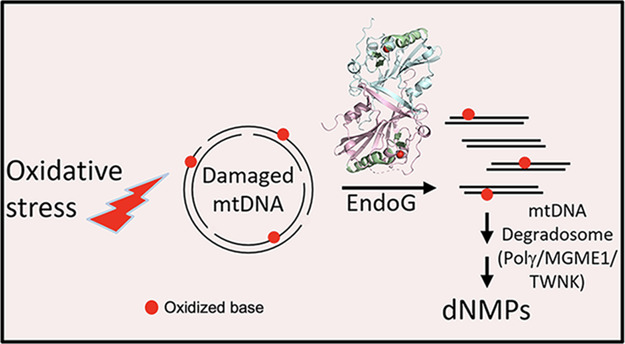

## Introduction

Endonuclease G (EndoG) was first identified and isolated from the mitochondria of rat liver and *Neurospora crassa*, revealing that it is a Mg^2+^-dependent endonuclease that cleaves phosphodiester bonds in nucleic acids to generate small fragments with 3′–OH and 5′-phosphate termini.^1,2^ Subsequent studies discovered similar mitochondrial endonucleases in *Saccharomyces cerevisiae*, mouse liver, chicken erythrocytes, and bovine heart cells.^3^ The enzyme isolated from chicken erythrocytes was named EndoG due to its preference for G-rich DNA sequences.^4^ EndoG can bind both DNA and RNA, showing a modest preference for single-stranded RNA (ssRNA) or DNA (ssDNA) with G-rich regions. Evolutionarily, EndoG is highly conserved in eukaryotes: human EndoG (hEndoG) shares 43, 54, and 94% sequence identity with *Caenorhabditis elegans*, *Drosophila melanogaster*, and mouse EndoG, respectively.^5^ EndoG is predominantly localized in mitochondria, where it is synthesized with an N-terminal mitochondrial localization sequence (MLS) that is subsequently cleaved to generate the mature form. Nevertheless, EndoG has also been detected in extranucleolar chromatin, indicating that a fraction of the protein functions outside the mitochondria.^6^

Crystal structures of EndoG from *D. melanogaster*, *C. elegans*, and mouse, with or without bound DNA, all reveal a homodimeric assembly, with each protomer containing a His-Me finger motif critical for DNA binding and cleavage.^7–9^ The His-Me finger motif consists of two β-strands and one α-helix, coordinating a central Mg^2+^ ion.^10^ This motif binds the minor groove of DNA, inducing conformational changes that widen and shallow the groove relative to canonical B-form DNA. In the structure of *C. elegans* EndoG (cEndoG) bound to ssDNA, the Mg^2+^ ion directly coordinates the scissile phosphate to form a Mg–O–P bond. A strictly conserved histidine on the first β strand in the His-Me finger motif acts as a general base to activate a water molecule, which attacks the phosphate, while a Mg^2+^-bound water protonates the leaving 3′-O atom, completing DNA cleavage and hydrolysis.^10^

EndoG was first postulated about three decades ago as possessing ribonuclease and RNase H activity, cleaving the RNA in RNA/DNA hybrids.^6^ It was shown then that EndoG specifically targeted mouse mitochondrial RNA and RNA/DNA substrates containing the origin of heavy-strand DNA replication. The cleavage sites identified in vitro corresponded to those observed in vivo, highlighting EndoG’s role in generating RNA primers essential for mitochondrial DNA replication by DNA polymerase γ (POLγ). Nevertheless, EndoG is most widely known for its role in nuclear DNA degradation during apoptosis, as reported in 2001.^11,12^ Nucleosomal DNA fragmentation is initiated by caspase-activated DNase (CAD) during apoptosis, but limited DNA fragmentation persists in CAD-deficient animals, indicating that another endonuclease plays a role in apoptotic DNA fragmentation. It was then demonstrated in *C. elegans* and mice that EndoG is translocated to the nucleus during apoptosis to induce nucleosomal DNA fragmentation.^12,13^ Additionally, EndoG has been identified as a key player in paternal mitochondrial elimination (PME), i.e., the process of degrading paternal mitochondrial DNA postfertilization, in *D. melanogaster* and *C. elegans* embryos.^14,15^ Together these studies indicate that EndoG targets nuclear and mitochondrial double-stranded DNA (dsDNA) during apoptosis and early embryogenesis.

In nonapoptotic cells, EndoG is also located within the mitochondrial matrix, where it is believed to be involved in removing oxidatively damaged mitochondrial DNA (mtDNA). Oxidative damage to mtDNA nucleobases can be repaired by base excision repair (BER).^16^ However, unlike nuclear DNA genomes having only two copies, hundreds to thousands of mtDNA molecules exist in most cells. Therefore, apart from mtDNA repair, eliminating defective mtDNA copies from the multicopy pool represents an alternative way of maintaining mtDNA integrity. It has been shown previously that mtDNA levels are diminished in wild-type mammalian cells upon H_2_O_2_-induced stress, but they remain unchanged in EndoG-deficient cells, implying that EndoG removes damaged mtDNA to maintain mtDNA integrity.^17,18^ Recent studies have further indicated that EndoG is involved in degrading mtDNA when oxidative lesions cannot be repaired efficiently, and that inhibition of BER can enhance mtDNA degradation in response to oxidative damage,^17,18^ strongly implying that these two events are interlinked and supporting the notion that irreparable mtDNA molecules are degraded. Oxidative stresses, such as high levels of reactive oxygen species (ROS), generate a variety of mtDNA lesions, including oxidized DNA bases, abasic sites, single-strand breaks (SSBs), and double-strand breaks (DSBs). EndoG preferentially cleaves DNA duplex near SSB sites in vitro,^19^ hinting that it might target oxidatively damaged mtDNA with SSBs.^20^ SSBs can also arise from cleavage of abasic lesions by the action of apurinic/apyrimidinic site endonuclease 1 (APE1), and from the erroneous or abortive activity of POLγ in BER or DNA replication pathways.^20^ These EndoG-cleaved linear mtDNA molecules may be further degraded by the mtDNA degradosome, comprising POLγ, MGME1, and TWNK helicase.^21^ *EndoG* deletion in mouse hearts caused mitochondrial dysfunction, high levels of ROS, a reduced mtDNA copy number, and cardiomyocyte hypertrophy, further supporting EndoG’s role in maintaining mtDNA levels and integrity.^22,23^

EndoG has also been identified as an endonuclease cleaving 5-hydroxymethylcytosine (5hmC)-modified DNA (5hmC-DNA), with a preference for hydroxy-methylated core sequences in the nucleus.^24^ This activity facilitates DSB formation and DNA recombination. Moreover, EndoG can cleave branched DNA structures, such as Holliday junctions (HJs), which are intermediate structures generated during homologous recombination and DNA repair.^25^ The cleavage of HJs by EndoG may contribute to resolving recombination intermediates and maintaining genomic stability, particularly under conditions of mitochondrial stress or during DNA repair processes.^25^ These findings highlight EndoG as a multifunctional endonuclease, crucial for maintaining genome integrity across cellular compartments.

Despite extensive studies, the nucleic acid targets of hEndoG and the molecular basis of its substrate preferences remain poorly understood. Here, we characterize the substrate preferences of hEndoG by assessing a broad range of nucleic acids, including ssDNA, ssRNA, dsDNA, RNA/DNA hybrid duplexes, 5hmC-DNA, and oxidatively damaged DNA, including nicked dsDNA, gapped dsDNA, and modified dsDNA harboring oxidized guanine (oxoG-DNA). We show that hEndoG displays a significant preference for cleaving oxidatively damaged DNA, including DNA with SSBs, i.e., gapped and nicked dsDNA, as well as oxoG-DNA. Our findings further evidence EndoG’s role in the first step of recognizing and cleaving oxidatively damaged DNA in the mtDNA degradation pathway responsible for maintaining mtDNA genome integrity.

## Materials and Methods

### Expression and Purification of hEndoG and the hEndoG-H141A Mutant

The cDNA of wild-type hEndoG was inserted into the pET28a­(+) vector and coexpressed with the *Drosophila melanogaster* EndoG inhibitor (dEndoGI) subcloned into the pETduet-1 vector. The H141A mutation was introduced into the pET28a­(+) plasmid using a QuikChange site-directed mutagenesis kit (Agilent), and the cDNA encoding F41 (*Salmonella* F41 fragment of Flagellin) was subcloned into the plasmid to express F41-fused cEndoG-H141A. Subsequently, these constructs were introduced individually into the BL21 (RIPL) bacterial strain of *E. coli*. Cell cultures were cultivated in LB Broth (Miller) medium. To express wild-type hEndoG, 50 μg/mL kanamycin sulfate (Sigma-Aldrich) and 100 μg/mL ampicillin sodium (Sigma-Aldrich) were added, and the cultures were incubated at 37 °C with agitation until the optical density (OD_600_) reached 0.8. For the expression of hEndoG-H141A, 50 μg/mL kanamycin sulfate (Sigma-Aldrich) was added to the cell cultures. Subsequently, the cultures were cooled to ∼20 °C, and then Isopropyl β-D-1 thiogalactopyranoside (Cyrusbioscience) was introduced to induce protein expression, with overnight shaking at 18 °C. Then the cell cultures were harvested, and the pellet mixture was dissolved using lysis buffer (50 mM Tris-HCl pH 7.4, 150 mM NaCl, 10 mM 2 mercaptoethanol). The cell pellets were further disrupted using a Microfluidics M-110P microfluidizer, and the mixture was centrifuged at 17,000 rpm at 4 °C for 30 min to collect the supernatant.

To purify wild-type hEndoG, the supernatant was applied to a HisTrap FF column (5 mL, GE Healthcare), followed by column washing with 6% buffer B (50 mM Tris-HCl pH 7.4, 150 mM NaCl, 10 mM 2-mercaptoethanol, 500 mM imidazole). The *D. melanogaster* EndoG inhibitor dEndoGI was eluted and removed during this step. The collected protein sample was subsequently subjected to another round of purification using a HiTrap Heparin HP column (5 mL, GE Healthcare). To further increase protein purity, a Superdex 75 Increase 10/300 GL column (GE Healthcare) was used sequentially. The final sample was stored in a gel filtration buffer consisting of 50 mM Tris-HCl (pH 7.4), 150 mM NaCl, and 1 mM dithiothreitol (DTT).

To purify hEndoG-H141A mutant protein, the supernatant was applied to a HisTrap FF column (5 mL, GE Healthcare), followed by column washing with 6% buffer B (50 mM Tris-HCl pH 7.4, 150 mM NaCl, 10 mM 2-mercaptoethanol, 500 mM imidazole). The protein was eluted under the condition of ∼ 24% buffer B. Collected fractions were then centrifuged, and the samples were dialyzed using TEV protease at 4 °C overnight to cleave the fusion protein (F41), employing a dialysis buffer (50 mM Tris-HCl pH 7.4, 150 mM NaCl, 10 mM 2-mercaptoethanol). The dialyzed protein sample was subjected to another round of purification using a HiTrap Heparin HP column (5 mL, GE Healthcare), before collecting the fractions containing the cleaved hEndoG-H141A. To further enhance protein purity, a Superdex 75 Increase 10/300 GL column (GE Healthcare) was used sequentially. The final sample was stored in a gel filtration buffer consisting of 50 mM Tris-HCl (pH 7.4), 150 mM NaCl, and 1 mM DTT.

### Measurement of the Dissociation Constant (K_D_) between hEndoG and Nucleic Acid Substrates

FAM-labeled substrates (10 nM, refer to Table 1 for sequences) were incubated with serially diluted catalytically inactive hEndoG-H141A mutant protein in a 40-μL reaction buffer consisting of 10 mM Tris-HCl pH 7.4, 10 mM NaCl, and 2.5 mM MgCl_2_ for 30 min. Fluorescence polarization signals were measured using a SpectraMax Paradigm Multi-Mode Microplate Reader (Molecular Devices) with an excitation wavelength at 485 nm and an emission wavelength at 535 nm. The fluorescence polarization value was determined by subtracting the value obtained when only DNA substrate was present. Data were fitted to specific binding according to a Hill slope equation using GraphPad Prism 9.0.

**Table 1:** DNA and RNA Oligonucleotides Used in the hEndoG DNA-Binding and Cleavage Assays^a^

oligo name	sequence
ssDNA	5′-TTAAGCCGAAGCTTATCGGTATCTCCTAACGCCAG AATTCGGCAGCGT-3′-FAM
dsDNA	5′-TTAAGCCGAAGCTTATCGGTATCTCCTAACGCCAGAATTCGGCAGCGT-3′-FAM
	3′-AATTCGGCTTCGAATAGCCATAGAGGATTGCGGTCTTAAGCCGTCGCA-5′
gapped dsDNA	5′-TTAAGCCGAAGCTTATCGGTATCTCCTAACGCCAGAATTCGGCAGCGT-3′-FAM
	3′-AATTCGGCTTCGAATAGCCATAGGGATTGCGGTCTTAAGCCGTCGCA-5′
nicked dsDNA	5′-TTAAGCCGAAGCTTATCGGTATCTCCTAACGCCAGAATTCGGCAGCGT-3′-FAM
	3′-AATTCGGCTTCGAATAGCCATAGAGGATTGCGGTCTTAAGCCGTCGCA-5′
ssRNA	5′-UUAAGCCGAAGCUUAUCGGUAUCUCCUAACGCCAG AAUUCGGCAGCGU-3′-FAM
RNA/DNA	5′-UUAAGCCGAAGCUUAUCGGUAUCUCCUAACGCCAGAAUUCGGCAGCGU-3′-FAM
	3′-AATTCGGCTTCGAATAGCCATAGAGGATTGCGGTCTTAAGCCGTCGCA-5′
DNA/RNA	5′-ACGCTGCCGAATTCTGGCGTTAGGAGATACCGATAAGCTTCGGCTTAA-3′-FAM
	3′-UGCGACGGCUUAAGACCGCAAUCCUCUAUGGCUAUUCGAAGCGAAUU-5′
5hmC-dsDNA	5′-TTAAGCCGAAGCTTATCGGTATCTCCTAACGCCAGAATTCGGCAGCGT-3′- FAM
	3′-AATTCGGCTTCGAATAGC**C**ATAGAGGATTGCGGTCTTAAGCCGTCGCA-5′
FAM-oxoG-dsDNA	5′-ACGCTGCCGAATTCTGGCGTTAG**G**AGATACCGATAAGCTTCGGCTTAA-3′- FAM
	3′-TGCGACGGCTTAAGACCGCAATCCTCTATGGCTATTCGAAGCCGAATT-5′
OxoG-dsDNA	5′-TTAAGCCGAAGCTTATCGGTATCTCCTAACGCCAGAATTCGGCAGCGT-3′- FAM
	3′-AATTCGGCTTCGAATAGCCATAGA**G**GATTGCGGTCTTAAGCCGTCGCA-5′

a Note: Modified nucleotides, i.e., 5hmC and 8-oxoG, are displayed in bold.

### Plasmid Nicking Assays to Measure hEndoG Nuclease Activity

pET28 plasmid DNA (100 ng) was incubated with hEndoG (0.4–600 nM) in 10 mM NaCl, 2 mM MgCl_2_ and 2 mM DTT at 37 °C for 5 min. The digested products were resolved on 1% agarose gels and stained with ethidium bromide, before destaining for 30 min. The digested products were detected by UV spectroscopy. The signal intensity of undigested plasmid DNA bands in the agarose gel was quantified using AlphaEaseFC software (version 4.0.0, Alpha Innotech) to calculate the percentage of degraded plasmid DNA.

### Measurement of hEndoG Activity on Various FAM-Labeled Nucleic Acids

Wild-type hEndoG (50 nM) was incubated with FAM (6-carboxyfluorescein)-labeled substrates (100 nM) in a 10-μL reaction buffer containing 10 mM Tris pH 7.4, 50 mM NaCl, 2 mM MgCl_2_, and 2 mM DTT at 37 °C. Oligonucleotide sequences were designed based on a previous study^24^ and are listed in Table 1. All the nucleic acid substrates were synthesized, purified, and purchased from MDBio Inc. Taiwan. Duplex DNA was prepared by annealing the FAM-labeled strand with its complementary strand. The mixture was heated at 95 °C for 5 min, followed by incubation at 30 °C for 10 min, and gradual cooling to room temperature, without additional PAGE purification. Cleavage reactions were stopped at 5 min by adding an equal amount of 2X TBE/urea sample buffer (BIO-RAD) and heating at 65 °C for 20 min. To completely release the FAM-labeled probe from the complementary strand, 2 μL of 100 μM competitive unlabeled DNA oligonucleotides was introduced. The resulting mixtures were initially heated at 95 °C for 5 min, followed by incubation at 30 °C for 10 min, before being cooled gradually to room temperature. Subsequently, the solutions were separated using a 20% denaturing acrylamide gel containing 6 M urea. FAM-labeled oligonucleotides, excited at 475 nm and emitted at 535 nm, were visualized in the resulting gels using a Typhoon FLA 9000 biomolecular imager (GE Healthcare Life Sciences). Band signal quantification was plotted using GraphPad Prism v. 9.0. Cleavage percentages were quantified based on the reduction of the FAM-labeled 48-nt substrate, normalized to the corresponding nucleic acid band in the no-enzyme control lane. All oligonucleotides were synthesized without 5′ phosphorylation unless otherwise noted.

### Construction of the Molecular Model of DNA-Bound hEndoG

To generate the hEndoG-ssDNA model, the crystal structure of cEndoG-ssDNA (PDB entry: 5GKP) was superimposed on the crystal structure of hEndoG-H141A/C113A (PDB entry: 9M0H) using PyMol. Doing so allowed the ssDNA to be positioned onto hEndoG. To construct the hEndoG-dsDNA model, the His-Me finger motif of the *Vibrio vulnificus* periplasmic endonuclease Vvn-dsDNA complex (PDB entry: 1OUP) was superimposed on that of hEndoG to determine the location of the dsDNA within the hEndoG-dsDNA complex using PyMol.

## Results

### hEndoG-H141A Mutant Protein Cannot Degrade Plasmid DNA

To determine the substrate preferences of hEndoG, first we expressed and purified both wild-type hEndoG and the hEndoG-H141A mutant protein. His141 in hEndoG is a catalytic residue that functions as a general base to activate a water molecule for nucleophilic attack on the scissile phosphate during DNA hydrolysis. As revealed by a previous study, mutation of this general base residue in *C. elegans* EndoG generates an inactive mutant enzyme capable of binding DNA but incapable of DNA cleavage.^5^ We coexpressed wild-type hEndoG with the *D. melanogaster* EndoG inhibitor (dEndoGI) to facilitate its soluble expression. To express the hEndoG-H141A mutant protein, its cDNA was fused to that of F41 (*Salmonella* F41 fragment of flagellin) to express a highly soluble F41-fused hEndoG-H141A peptide, and then the N-terminal F41 tag was cleaved away during protein purification steps. We purified both proteins as homodimers to high homogeneity by chromatographic methods with a molecular weight of ∼50 kDa, as confirmed by gel filtration profiles and SDS-PAGE (Figure 1).

**Figure 1:**
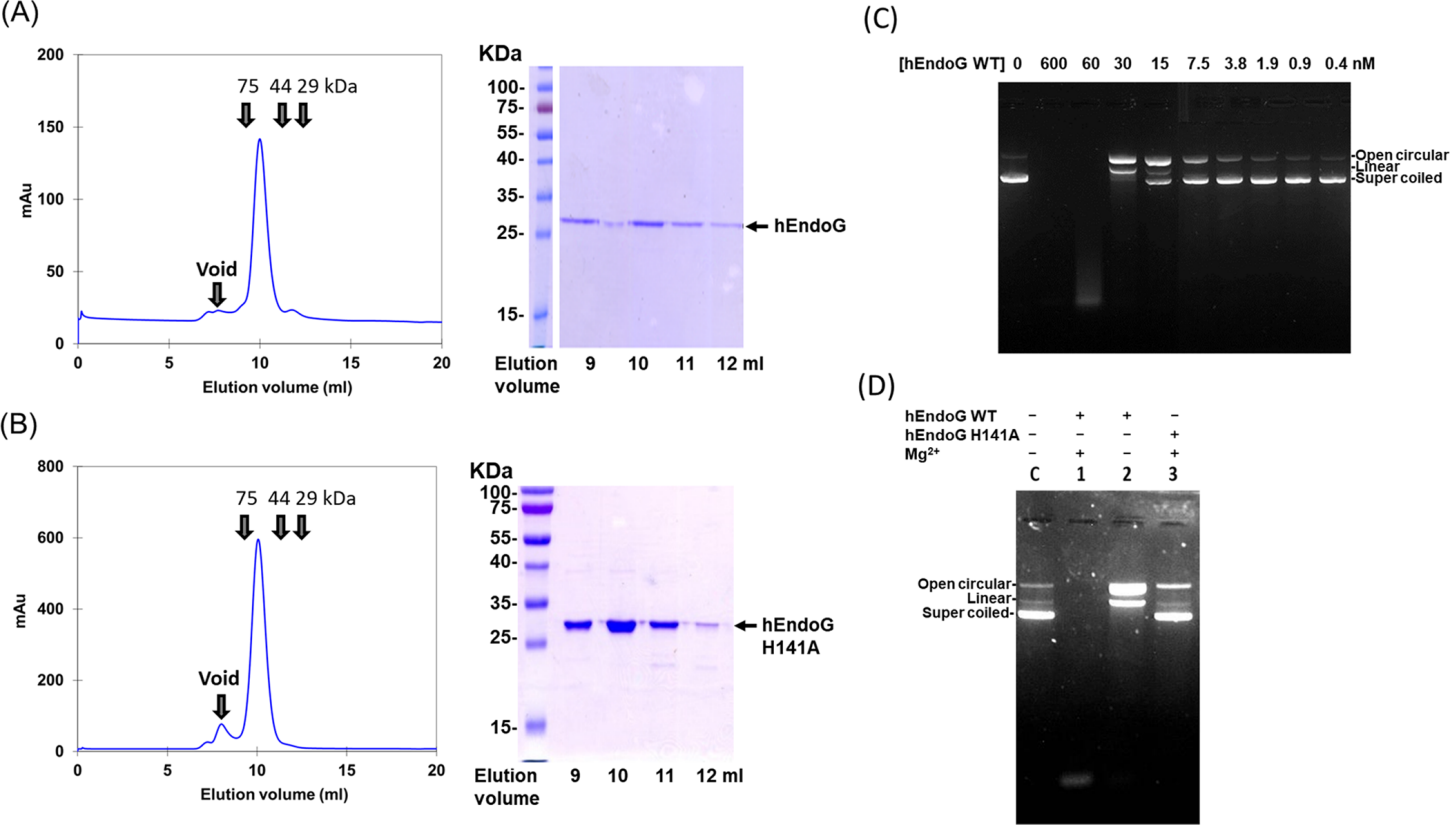
Active recombinant hEndoG and inactive hEndoG-H141A mutant in cleaving plasmid DNA. (A, B) Panels at left show the elution profiles of wild-type hEndoG and the hEndoG-H141A mutant from the Superdex 75 Increase 10/300 GL column. The protein markers used for calibration are conalbumin (75 kDa), ovalbumin (44 kDa), and carbonic anhydrase (29 kDa), as indicated by arrows. Both hEndoG and hEndoG-H141A eluted as homodimers with a molecular weight of ∼50 kDa. The panels at right display the SDS-PAGE of the eluted fractions. The elution volume corresponds to the fractions collected under the peaks shown in the panels at left. (C) pET28 plasmid DNA (100 ng) was incubated with purified hEndoG (0.4–600 nM) in 10 mM NaCl, 2 mM MgCl_2_, and 2 mM DTT at 37 °C for 5 min. The digested products were resolved on 1% agarose gels, showing that the supercoiled DNA was cleaved into open circular and linear forms. (D) Plasmid DNA (100 ng) was incubated with either hEndoG (1 μM) or hEndoG-H141A (1 μM) in the presence or absence of Mg^2+^ ions.

Next, we evaluated the endonuclease activity of hEndoG by conducting plasmid nicking assays using different concentrations of the enzyme while retaining a constant plasmid DNA concentration. The plasmid DNA primarily adopted a supercoiled conformation, which was converted to an open circular configuration by a single nick or into linear DNA following double nicks by hEndoG within the supercoiled DNA structure. Ultimately, the plasmid DNA became completely fragmented with increasing hEndoG concentrations (Figure 1C). In comparison to wild-type hEndoG enzyme that degraded the supercoiled DNA, the hEndoG-H141A mutant hardly degraded plasmid DNA in the presence of cofactor Mg^2+^ ions, supporting that mutation of catalytic residue His141 to Ala abolishes the endonuclease activity of hEndoG (Figure 1D).

### hEndoG-H141A Binds with Similar Affinities to a Range of Substrates

Next, we investigated the nucleic acid binding affinity of hEndoG using the inactive hEndoG-H141A mutant as it could not cleave nucleic acid substrates. A range of nucleic acids were synthesized, including ssDNA, dsDNA, nicked dsDNA, gapped dsDNA, ssRNA, RNA/DNA hybrid duplex, oxoG-dsDNA and 5hmC-dsDNA, all with a length of ∼ 48 nucleotides (Table 1). To evaluate the binding affinity between the inactive hEndoG-H141A mutant and these various DNA and RNA substrates, we synthesized 6-carboxyfluorescein (FAM)-labeled nucleic acid substrates and then measured the equilibrium dissociation constant (*K*_D_) between hEndoG-H141A and each nucleic acid substrate by monitoring the change in fluorescence polarization (FP) signal upon protein-nucleic acid interactions. We found that hEndoG-H141A bound with similar affinities in the μM range to unmodified DNA substrates, including ssDNA (*K*_D_ = 2.4 ± 0.6 μM), dsDNA (*K*_D_ = 3.3 ± 0.6 μM), nicked dsDNA (*K*_D_ = 3.2 ± 1.3 μM), and gapped dsDNA (*K*_D_ = 2.9 ± 0.8 μM) (Figure 2A). hEndoG-H141A bound ssRNA with a similar affinity (*K*_D_ = 3.1 ± 0.7 μM) to ssDNA, i.e., in the μM range, yet it bound the RNA/DNA hybrid duplex with a ∼10-fold reduced affinity (*K*_D_ = 31.3 ± 19.4 μM) (Figure 2B). Performing FAM labeling at the 3′ end of either the RNA or DNA strand of the hybrid duplex did not impact the binding affinity, with respective *K*_D_ values of ∼31–42 μM (Figure 2B). hEndoG-H141A bound modified DNA substrates with comparable affinities in the μM range, including FAM-oxoG-dsDNA (*K*_D_ = 6.0 ± 2.4 μM), oxoG-dsDNA (*K*_D_ = 10.6 ± 3.4 μM), and 5hmC-dsDNA (*K*_D_ = 2.3 ± 6.1 μM) (Figure 2C). Collectively, these results reveal that hEndoG-H141A does not present a clear preference for binding with any of these nucleic acid substrates, encompassing single-stranded and double-stranded DNA, as well as intact, nicked, gapped and modified dsDNA. hEndoG-H141A variant selectively disfavors interactions with RNA/DNA hybrid duplexes, which adopt an A-form conformation characterized by a shallow and narrow minor groove. This geometry may introduce steric hindrance that impedes proper accommodation of the His-Me finger motif. Collectively, these findings indicate that hEndoG preferentially interacts with DNA substrates, whereas RNA/DNA hybrids are unlikely to represent its favored binding substrates.

**Figure 2:**
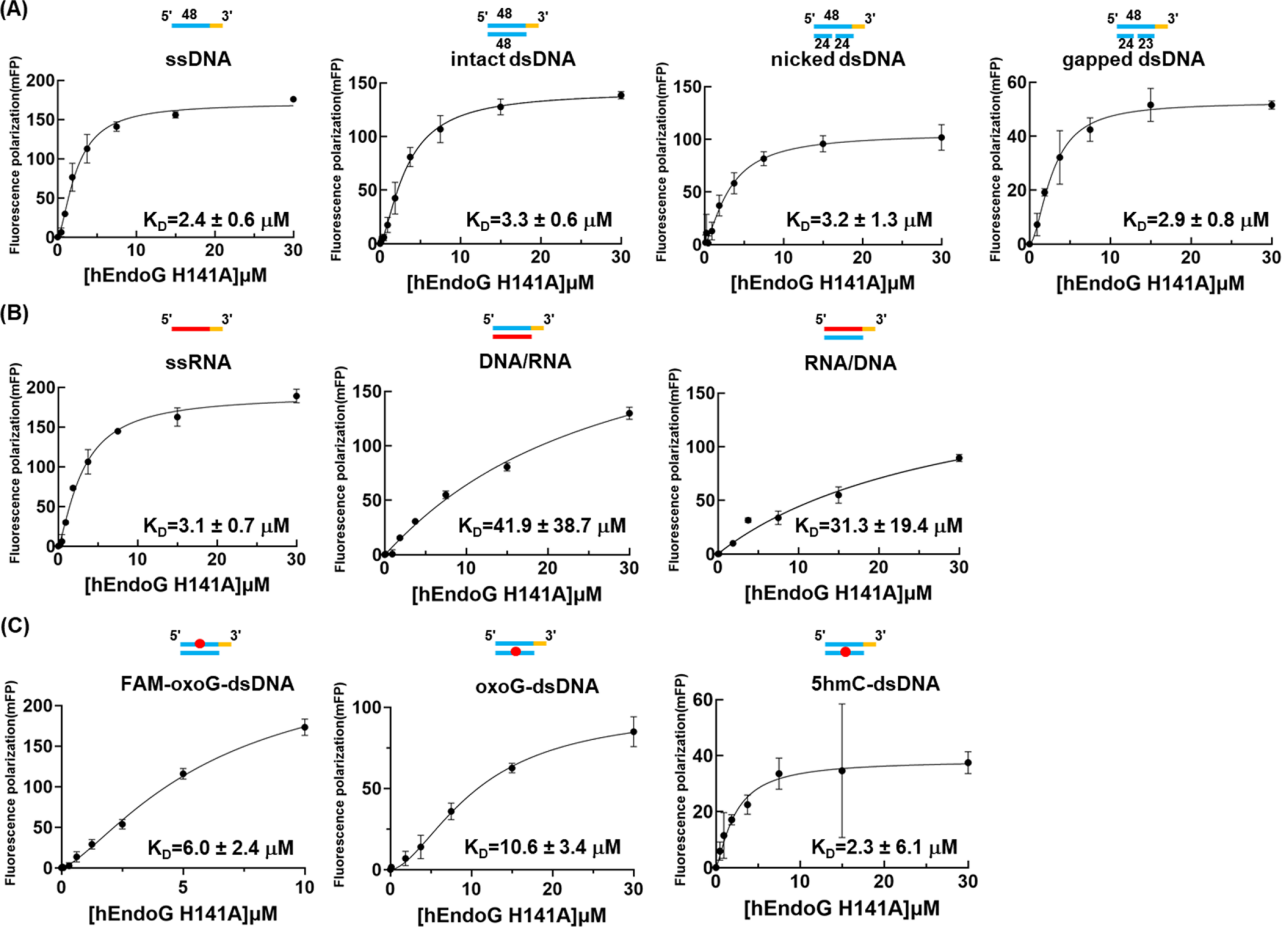
hEndoG-H148A binds a range of nucleic acid substrates with similar affinities. (A) Binding affinities between the hEndoG-H148A mutant and DNA substrates, including ssDNA, intact dsDNA, nicked dsDNA, and gapped dsDNA. (B) Binding affinities between the hEndoG-H148A mutant and RNA substrates, including ssRNA and RNA/DNA hybrids. (C) Binding affinities between the hEndoG-H148A mutant and modified dsDNA substrates, including oxoG-dsDNA and 5hmC-dsDNA. The dissociation constants (*K*_D_) were measured by fluorescence polarization using FAM-labeled substrates. The error bars in each plot represent mean ± SD from three independent experiments.

### hEndoG Preferentially Cleaves Nicked and Gapped dsDNA

To investigate the preferred cleavage substrates of hEndoG, we compared the cleavage patterns generated by hEndoG upon its incubation with ssDNA, intact dsDNA, nicked DNA and gapped DNA. We observed ladder-like cleavage patterns for hEndoG upon encountering ssDNA and dsDNA, indicating that the enzyme cleaves both substrates nonspecifically (Figure 3A). However, hEndoG generated a major cleavage site at around the 22nd nucleotide (nt) of nicked and gapped DNA substrates on the opposite strand of the nicked or gapped site (Figure 3A, major cleavage sites indicated by red triangles). Comparison of the degradation patterns shows that hEndoG preferentially cleaves gapped and nicked DNA on the opposite strand of the nicked or gapped site relative to intact dsDNA substrate (Figure 3A, Right). We also compared hEndoG activity in cleaving ssRNA and DNA/RNA hybrid substrates. We found that ssRNA was strongly cleaved in a nonspecific manner, whereas the FAM-labeled RNA or DNA within DNA/RNA hybrid duplexes was barely cleaved (Figure 3B).

**Figure 3:**
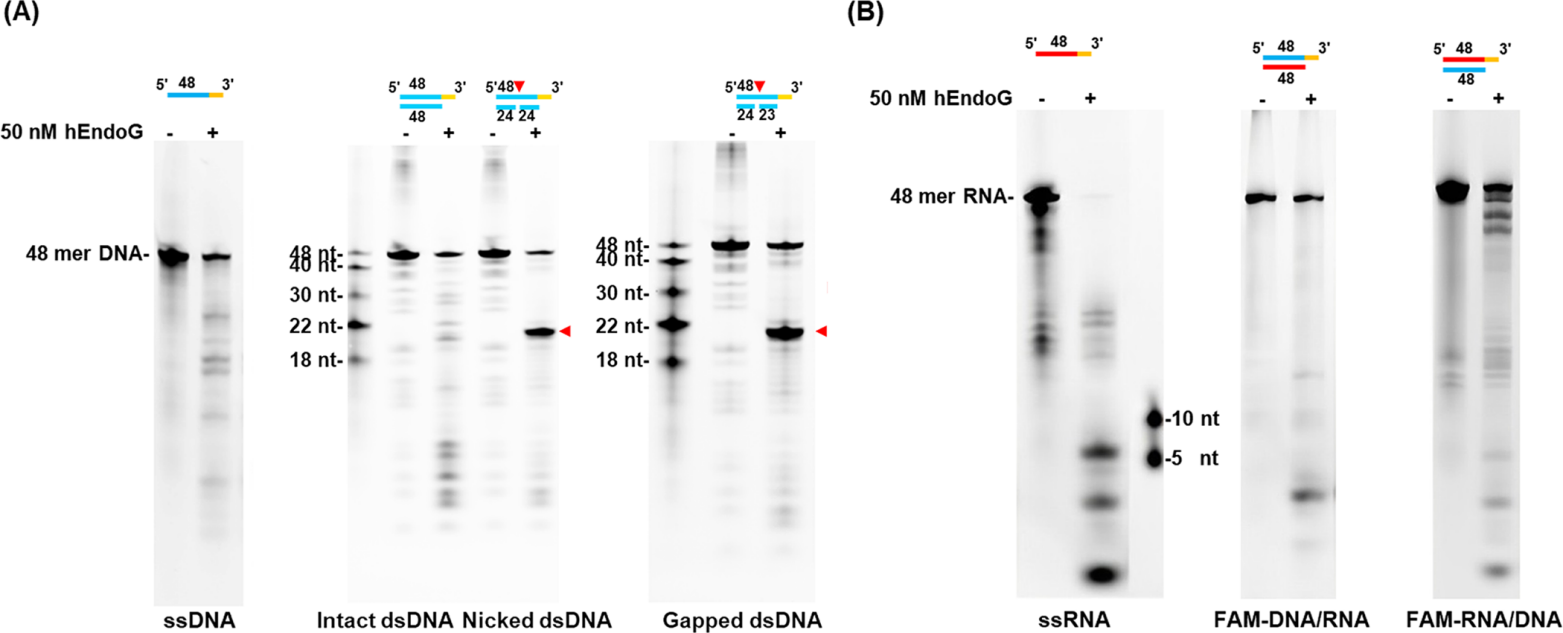
hEndoG preferentially cleaves the strand opposite to gaps and nicks in dsDNA substrates. (A) Left: Nuclease activity assays of hEndoG (50 nM) degrading the 3′-FAM-labeled 48 basepair (bp) ssDNA, intact dsDNA, gapped dsDNA and nicked dsDNA (100 nM) for the same timespan (5 min) at 37 °C. Different DNA size markers are shown along the left side of the gel. (B) Left: Nuclease activity assays of hEndoG (50 nM) degrading the 3′-FAM-labeled 48-bp ssRNA, as well as DNA/RNA hybrid duplexes in which FAM has been labeled on either the RNA or DNA strand (100 nM), over the same timespan (5 min) at 37 °C.

In terms of modified dsDNA substrates, we found that both 8oxoG-dsDNA and 5hmC-dsDNA were preferentially cleaved at the opposite strand of the modified sites (Figure 4A). However, the strand harboring the modified sites, i.e., FAM-oxoG-dsDNA and FAM-5hmC-dsDNA, were less cleaved (Figure 4B). For an unknown reason, hEndoG specifically generated ∼5-nt products upon cleaving FAM-8oxoG-dsDNA (Figure 4B). For 5hmC-dsDNA, cleavage products migrated as smaller, low-intensity fragments that were less readily detected by fluorescent scanning. Overall, these results reveal that hEndoG preferentially cleaves both ssDNA and ssRNA, as well as the opposite strands of dsDNA containing a nick, gap, 5oxoG or 5hmC.

**Figure 4:**
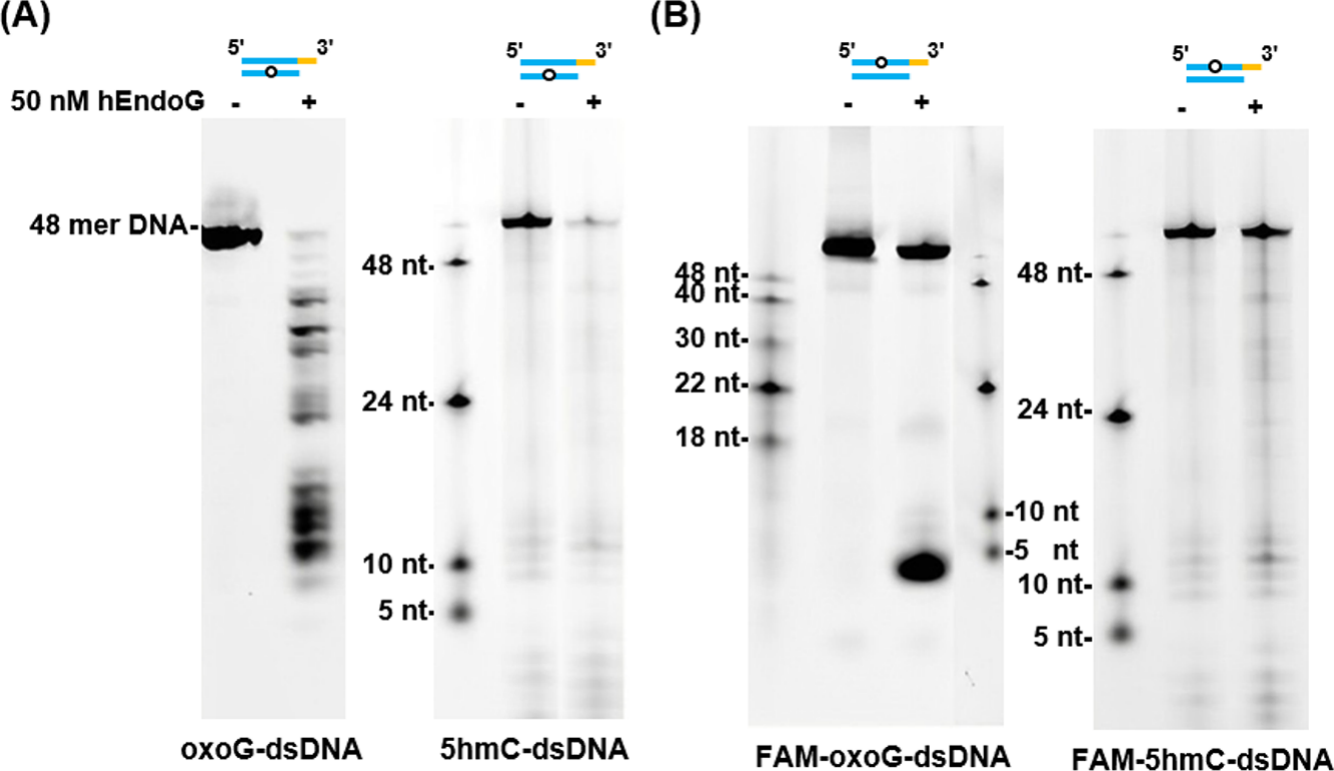
hEndoG preferentially cleaves the strand of dsDNA opposite a modified base. (A) hEndoG (50 nM) preferentially cleaves dsDNA substrates containing a modified base, such as oxoG-dsDNA or 5hmC-dsDNA (100 nM). The FAM fluorophore was attached to the 3′ end of the unmodified strand of the dsDNA substrate. (B) hEndoG cleaves less efficiently the FAM-labeled strand containing modified bases of the FAM-oxoG-dsDNA and FAM-5hmC-dsDNA substrates.

### Structural Models of hEndoG Bound with DNA

Due to the difficulty in obtaining cocrystals of DNA-bound hEndoG, we constructed structural models of hEndoG-DNA complexes to investigate the possible basis for hEndoG’s cleavage preferences. The structural model of hEndoG bound to ssDNA was generated by superimposition of the crystal structures of hEndoG (PDB entry: 9M0H) onto the *C. elegans* cEndoG-ssDNA complex (PDB entry: 5GKP).^8^ The ssDNA adopted from the cEndoG-ssDNA structure is well positioned in relation to hEndoG, with a Mg^2+^ ion located close (2.0 Å) to the scissile phosphate oxygen atom (O–Mg^2+^) (Figure 5A).

**Figure 5:**
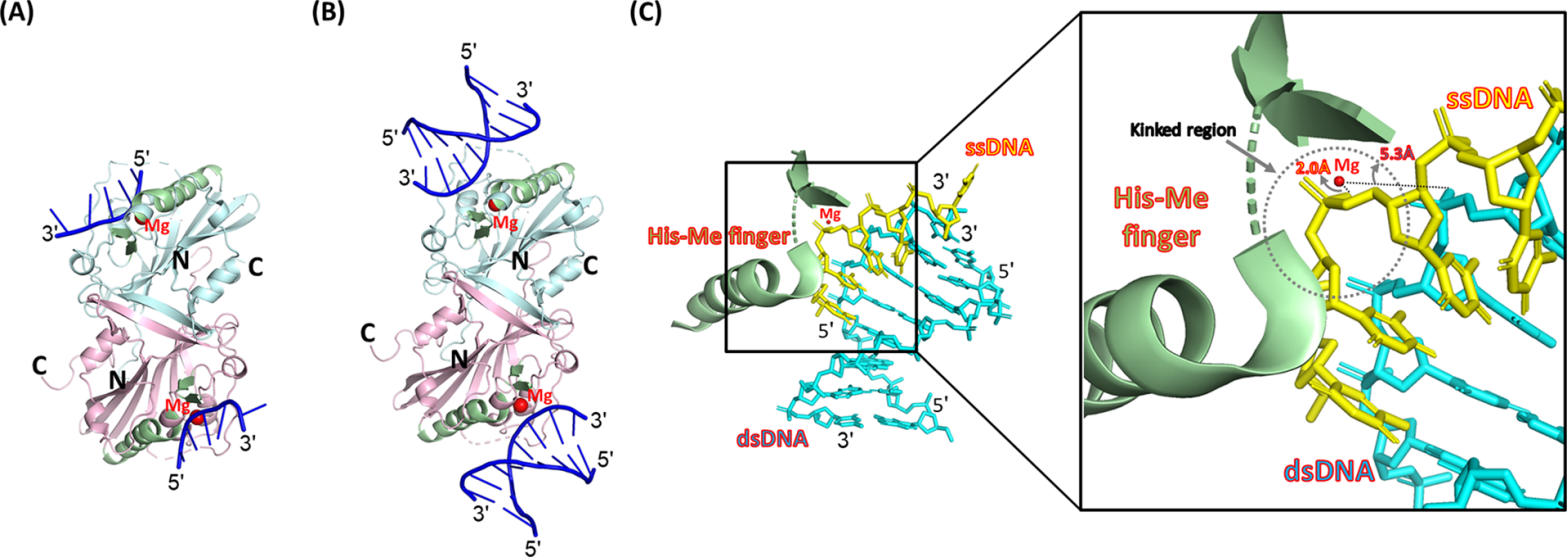
Structural models of hEndoG bound to ssDNA and dsDNA. (A) Structural model of the hEndoG-ssDNA complex. The ssDNA was adopted from the crystal structure of the cEndoG-ssDNA complex (PDB id: 5GKP). (B) Structural model of the hEndoG-dsDNA complex. The dsDNA was adopted from the Vvn-DNA complex (PDB id: 1OUP). The chain A and B of the dimeric hEndoG is colored in blue and pink, respectively. (C) The superimposed ssDNA (colored in yellow) vs ssDNA (in cyan) against the His-Me finger in which the magnesium ion (marked in red) forms polar contacts with the oxygen atom of the scissile phosphate of the ssDNA and dsDNA at a distance of 2.0 and 5.3 Å, respectively. A kinked region was indicated by a broken circle on the ssDNA.

Next, we constructed a structural model of hEndoG bound to dsDNA by superimposing the His-Me finger motif of hEndoG with the one embedded in the Vvn-dsDNA complex (PDB entry: 1OUP).^26^ Our structural model of the hEndoG-dsDNA complex reveals that the dsDNA is bound next to the His-Me finger motif, with a O–Mg^2+^ distance of 5.3 Å (Figure 5B). Close inspection of the superimposed active site of our hEndoG-DNA models reveals that the more bent conformation of the ssDNA allows it to interact more closely with the active site compared to the dsDNA (Figure 5C). This observation implies that EndoG likely preferentially cleaves ssDNA with a kinked structure bearing a scissile phosphate that is positioned close to the catalytic Mg^2+^ ion. Presumably, gapped and nicked dsDNA can also adopt this kinked structure, and so are preferentially cleaved by hEndoG. Collectively, our modeling analysis supports that ssDNA, and possibly nicked and gapped dsDNA, that form kinked structures are preferred substates for cleavage by hEndoG (Figure 5C).

## Discussion

EndoG is widely known as an apoptotic endonuclease that degrades nuclear DNA in a nonspecific manner during apoptosis.^2^ Beyond its well-established role in apoptosis, it has been shown previously that EndoG depletion can reduce mtDNA copy number, and that EndoG is related to the removal of oxidized mitochondrial DNA.^3,4^ Recent studies have also unveiled additional biological functions for EndoG, such as an ability to activate autophagy by competitively binding with 14–3–3 protein in the cytoplasm and cleaving 5hmC-modified DNA to induce DNA recombination and DNA damage responses in the nucleus.^24,27^ However, it had been unclear if EndoG preferentially cleaves nucleic acid substrates in these processes. Therefore, the aim of our study was to explore the endonuclease activity in vitro of hEndoG in terms of binding and degradation of a group of potential substates, with a view to illuminating EndoG’s various functions.

To do so, we prepared a variety of DNA and RNA substrates to explore the diverse biological functions of hEndoG. To investigate its role in RNA primer generation during mtDNA replication, we prepared RNA/DNA hybrid duplexes in which the fluorescent FAM probe was labeled either at the 3′ end of the RNA or DNA. We found that hEndoG binds and cleaves RNA/DNA hybrid duplexes with an approximately 10-fold lower activity relative to ssRNA, ssDNA, and nicked or gapped dsDNA substrates. These results indicate that hEndoG is likely not a specific endonuclease that cleaves RNA/DNA duplex at the G-rich region in mtDNA to generate RNA primers, as proposed previously.^6^

To investigate the role of hEndoG in removing oxidatively damaged DNA, we prepared nicked, gapped, and 8oxoG-modified dsDNA substrates. We observed similar μM-level binding affinities for hEndoG with intact dsDNA, nicked dsDNA, gapped dsDNA, and 8oxoG-modified dsDNA. However, our nuclease activity assays revealed distinctive DNA degradation patterns for these substrates, providing evidence that hEndoG preferentially cleaves at the opposite strand of the gapped or nicked site and the oxoG-containing strand. These findings support a previous suggestion that EndoG participates in removing oxidized DNA under conditions of oxidative stress.^17^ Our structural model of hEndoG bound to ssDNA further shows that these DNA substrates can adopt kinked structures, with a scissile phosphate located close to the Mg^2+^ ion in the His-Me finger motif (Figure 5C). These types of substates—including ssDNA, nicked dsDNA, and gapped dsDNA-represent ROS-damaged DNA and so could be preferentially cleaved by hEndoG (Figure 6). Similarly, DNA containing the 5hmC epigenetic modification and G-quadruplexes may also bear kinked structures that could be preferentially cleaved by hEndoG.^24,28^ The substrates used here were not enriched for such motifs, which may explain differences in cleavage intensity relative to earlier studies.^24,28^

**Figure 6:**
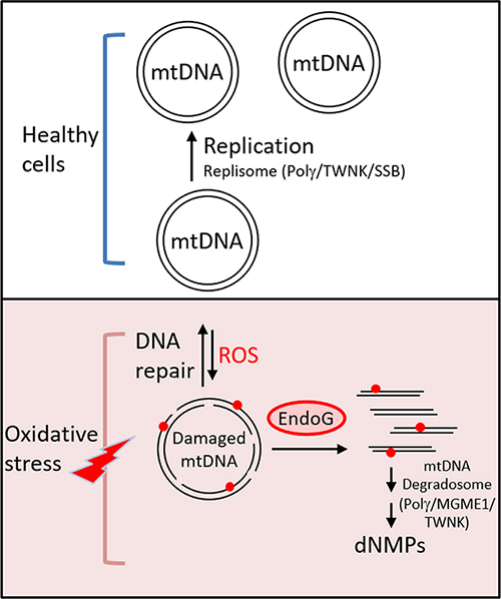
Proposed model of how hEndoG cleaves oxidatively damaged mtDNA to generate linear mtDNA intermediates under oxidative stress in the mtDNA degradation pathway. In healthy cells, mtDNA is replicated by the replisome comprising POLγ, TWNK, and SSB. Under oxidative stress, mtDNA replication is stalled by the presence of ROS-damaged mtDNA containing SSBs and oxidized bases (indicated by red dots). EndoG subsequently degrades these oxidatively damaged mitochondrial DNA molecules to generate linear DNA products. The fragmented linear DNA molecules are further degraded into mononucleotides by the mitochondrial DNA degradosome.

## Conclusions

Our findings indicate that oxidative lesions (especially 8-oxoG) can create structural distortions sufficient to elicit strong cleavage even in the absence of G-rich contexts. In summary, our biochemical results support a model by which oxidatively damaged mtDNA containing SSBs and oxidized bases is preferentially cleaved by hEndoG to generate linear DNA fragments. These linear DNA molecules can then be further degraded by the mtDNA degradosome, comprising POLγ, MGME1, and TWNK helicase^21,29–31^ (Figure 6). Thus, hEndoG is an important endonuclease targeting oxidatively damaged DNA and it is responsible for safeguarding mitochondrial genome integrity.

## Notes

UniProtKB Q14249 is the accession number for human Endonuclease G.
